# Genetic counseling for pre-implantation genetic testing of monogenic disorders (PGT-M)

**DOI:** 10.3389/frph.2023.1213546

**Published:** 2023-12-15

**Authors:** Firuza Parikh, Arundhati Athalye, Prochi Madon, Meenal Khandeparkar, Dattatray Naik, Rupesh Sanap, Anuradha Udumudi

**Affiliations:** ^1^ Department of Assisted Reproduction and Genetics, Jaslok-FertilTree International Fertility Centre, Jaslok Hospital and Research Centre, Mumbai, India; ^2^ GeneTech Laboratory, Banjara Hills, Hyderabad, India

**Keywords:** PGT, genetic counseling, preimplantation genetic testing, PGT-M, monogenic disorders, GC for PGT-M, Mendelian disorders

## Abstract

Pre-implantation genetic testing (PGT) is a vital tool in preventing chromosomal aneuploidies and other genetic disorders including those that are monogenic in origin. It is performed on embryos created by intracytoplasmic sperm injection (ICSI). Genetic counseling in the area of assisted reproductive technology (ART) has also evolved along with PGT and is considered an essential and integral part of Reproductive Medicine. While PGT has the potential to prevent future progeny from being affected by genetic conditions, genetic counseling helps couples understand and adapt to the medical, psychological, familial and social implications of the genetic contribution to disease. Genetic counseling is particularly helpful for couples with recurrent miscarriages, advanced maternal age, a partner with a chromosome translocation or inversion, those in a consanguineous marriage, and those using donor gametes. Partners with a family history of genetic conditions including hereditary cancer, late onset neurological diseases and with a carrier status for monogenic disorders can benefit from genetic counseling when undergoing PGT for monogenic disorders (PGT-M). Genetic counseling for PGT is useful in cases of Mendelian disorders, autosomal dominant and recessive conditions and sex chromosome linked disorders and for the purposes of utilizing *HLA* matching technology for creating a savior sibling. It also helps in understanding the importance of PGT in cases of variants of uncertain significance (VUS) and variable penetrance. The possibilities and limitations are discussed in detail during the sessions of genetic counseling.

## Introduction

Pre-implantation genetic testing (PGT) is vital in preventing chromosomal aneuploidies and other genetic disorders including rare genetic monogenic conditions in human embryos created by intracytoplasmic sperm injection (ICSI). The utility of PGT in infertility management has steadily gained importance over the last two decades. Although the practice of genetic counseling originated in the 1960s along with the emergence of prenatal testing, genetic counseling in the area of assisted reproductive technology (ART) has evolved as an integral part of the PGT process and is widely used in reproductive medicine (1).

Genetic counseling is defined by the National Society of Genetic Counselors, USA, as a process of helping people understand and adapt to the medical, psychological and familial implications of the genetic contributions to disease. This process integrates the following: (a) Interpretation of the family and medical history to assess the chance of disease occurrence or recurrence. (b) Education about inheritance, testing, management, prevention, available resources and current research. (c) Counseling to facilitate informed choices and adaptation to the risk or condition (2, 3). Genetic counseling is specially helpful for families facing complex situations that may arise during the process of PGT for Monogenic disorders (PGT-M). The current review is an attempt to introduce readers to some situations that can arise while offering PGT-M.

While the advancement of diagnostic technologies offers prevention of genetic disorders and birth defects, the dissemination and interpretation of information on the disease-causing variations and their solutions can best be served with genetic counseling. With PGT emerging as an early alternative to post-conception prenatal diagnosis, couples are in a better position to plan a pregnancy with the help of *in vitro* fertilization (IVF) driven PGT. Preimplantation genetic technology aims to prevent the trauma of repeated termination of pregnancies in cases where genetic abnormalities in the fetus are diagnosed much later by prenatal testing. Genetic counseling plays an important role in communicating the benefits, limitations and complexities of PGT in an IVF set up in a non-directive way, with empathy.

## Counseling during the PGT procedure

PGT is an integral part of an experienced IVF centre and is done in collaboration with a genetics laboratory with skills to accurately report on the results of testing 1–8 embryonic cells. On day 5–6 after the ICSI procedure, about 5–8 herniated trophectoderm cells are biopsied from a site on the blastocyst away from the inner cell mass by an experienced embryologist. The biopsied cells from each embryo are transferred into separate tubes and sent to specialized genetic laboratories for testing (4, 5). All concerns of patients related to the invasive nature of micromanipulation and lack of long-term safety outcome data should be addressed with transparency. Reassurance about the safety of recent biopsy techniques of the trophectoderm compared to previously used techniques should be shared (6). After biopsy, the embryos are vitrified until the availability of results. Although the risks associated with thawing of vitrified embryos is minimal, couples should be made aware of them (7).

PGT which was previously called pre-implantation genetic diagnosis (PGD) includes three terms, PGT-A, PGT-SR and PGT-M (8–10). PGT screens embryos for chromosomal aneuploidies (PGT-A) thereby increasing the chances of implantation and reducing pregnancy loss (11). PGT for structural rearrangement (PGT-SR) including reciprocal balanced translocations, inversions or insertions is helpful for individuals with chromosomal rearrangements. PGT for monogenic diseases (PGT-M) has the potential to offer reproductive options to couples who are at an increased risk of having progeny with single gene disorders such as thalassemia, sickle cell anemia, cystic fibrosis and Huntington disease to name a few. PGT-A is also carried out together with PGT-M to increase the chances of success, by selecting euploid unaffected embryos for transfer. This review is limited to the discussion of PGT-M.

## Pre-implantation genetic testing for monogenic disorders (PGT-M)

In 1990, Handyside's group first reported live births using IVF followed by PGD for an X-linked disorder (12). After 2005, different methodologies evolved resulting in a plethora of techniques capable of diagnosing genetic disorders. These were quickly adopted in PGT. They include haplotyping (13), comparative genomic hybridization (aCGH) (14), next generation sequencing (NGS) (15), karyomapping (16) and linkage analysis (17). PGT-M is presently available for identifying pathogenic or likely pathogenic disease causing variants in embryos for all known monogenic disorders (18). These disorders encompass both common Mendelian conditions such as beta thalassemia, cystic fibrosis, hemophilia, sickle cell anemia, Huntington's chorea, Duchenne muscular dystrophy, as well as rarer genetic diseases like methylmalonic acidemia and lysosomal storage disorders. Additionally, specific mitochondrial disorders like Leigh syndrome where nuclear DNA is involved (19, 20), are also included. The aim of genetic counseling is to effectively convey the advantages and constraints of PGT while ensuring that patients have comprehensive information to make an informed decision, taking into account the outcomes derived from the test. Some of the important features of PGT-M genetic counseling are discussed in this review.

## Pre-PGT-M genetic counseling

It is important for all couples considering PGT-M to participate in genetic counseling and share their family history, medical records and results of molecular studies carried out for family members. A prerequisite for PGT-M testing is that the disease-causing variant is identified and classified as per American College of Medical Genetics (ACMG) guidelines for pathogenicity before initiating PGT-M (21–23). Based on the pedigree analysis and review of reports, the genetic counselor is in a position to discuss the inheritance pattern of the condition in the family, risks to the offspring and available preventive options including prenatal or pre-implantation genetic testing. The benefits of PGT vs. its risks, limitations, and cost of testing should be communicated to the family. Informed written consent is necessary and should be taken prior to initiating the PGT procedure.

## Determination of the disease causing variation and evaluation of the inheritance pattern are prerequisites for PGT-M

**Figure 1:**
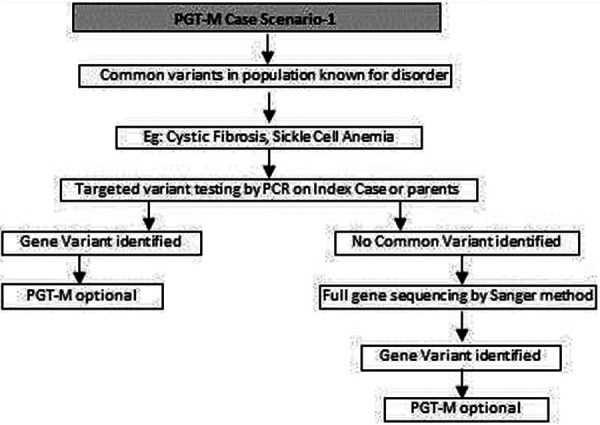
Genetic diagnostic workflow for PGT-M of common known variants in certain disorders.

**Figure 2:**
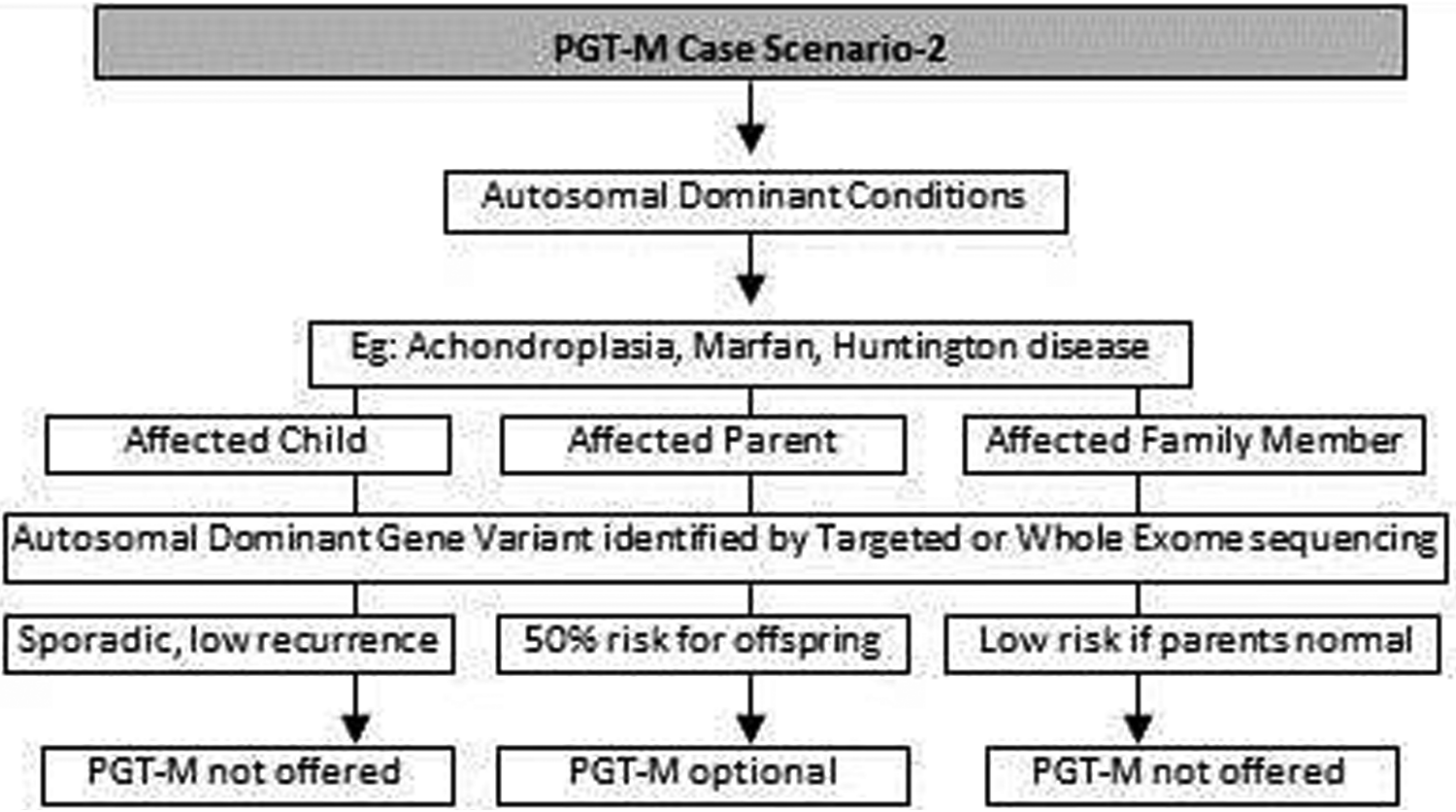
Genetic diagnostic workflow for PGT-M of autosomal dominant conditions.

**Figure 3:**
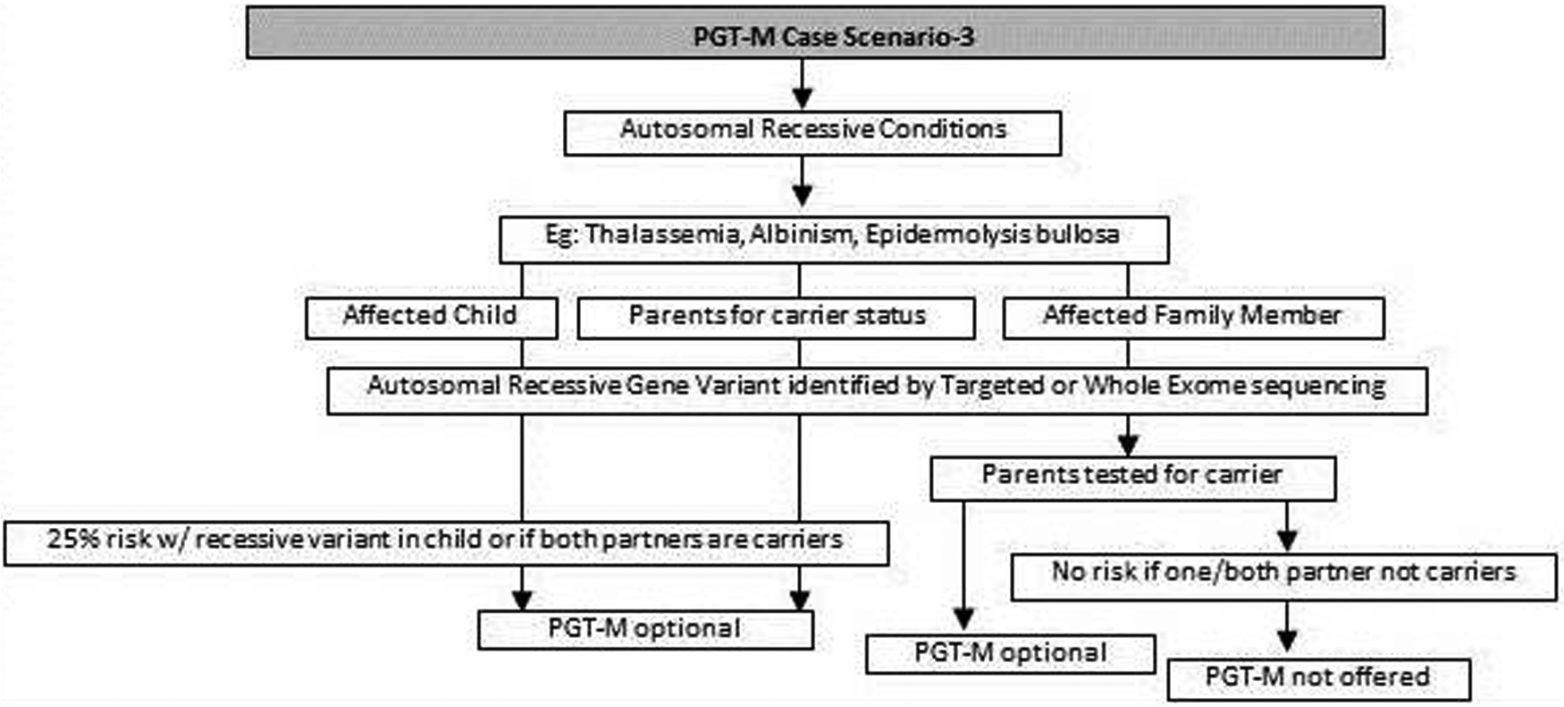
Genetic diagnostic workflow for PGT-M of autosomal recessive conditions.

**Figure 4:**
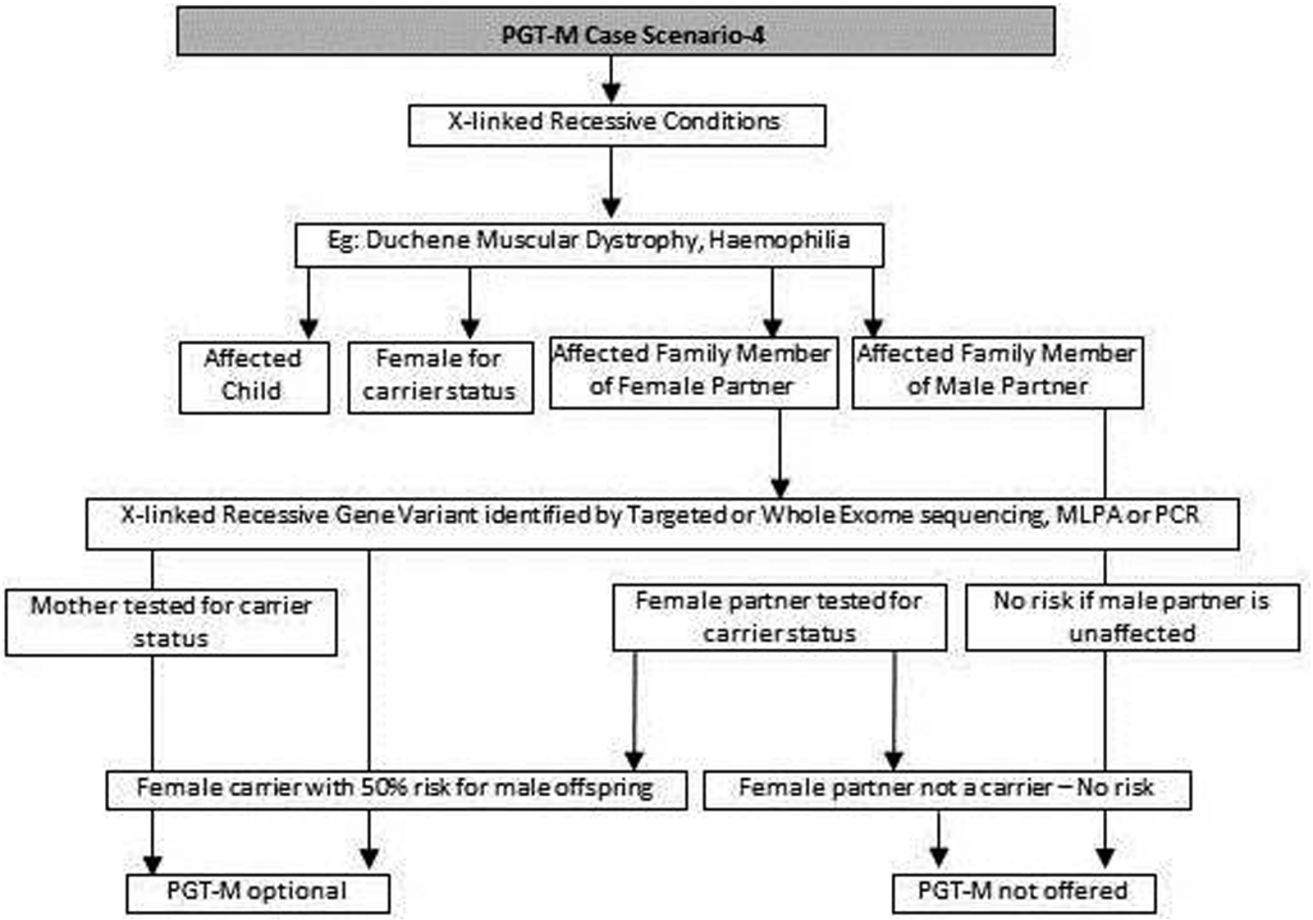
Genetic diagnostic workflow for PGT-M of X-linked recessive conditions.

**Figure 5:**
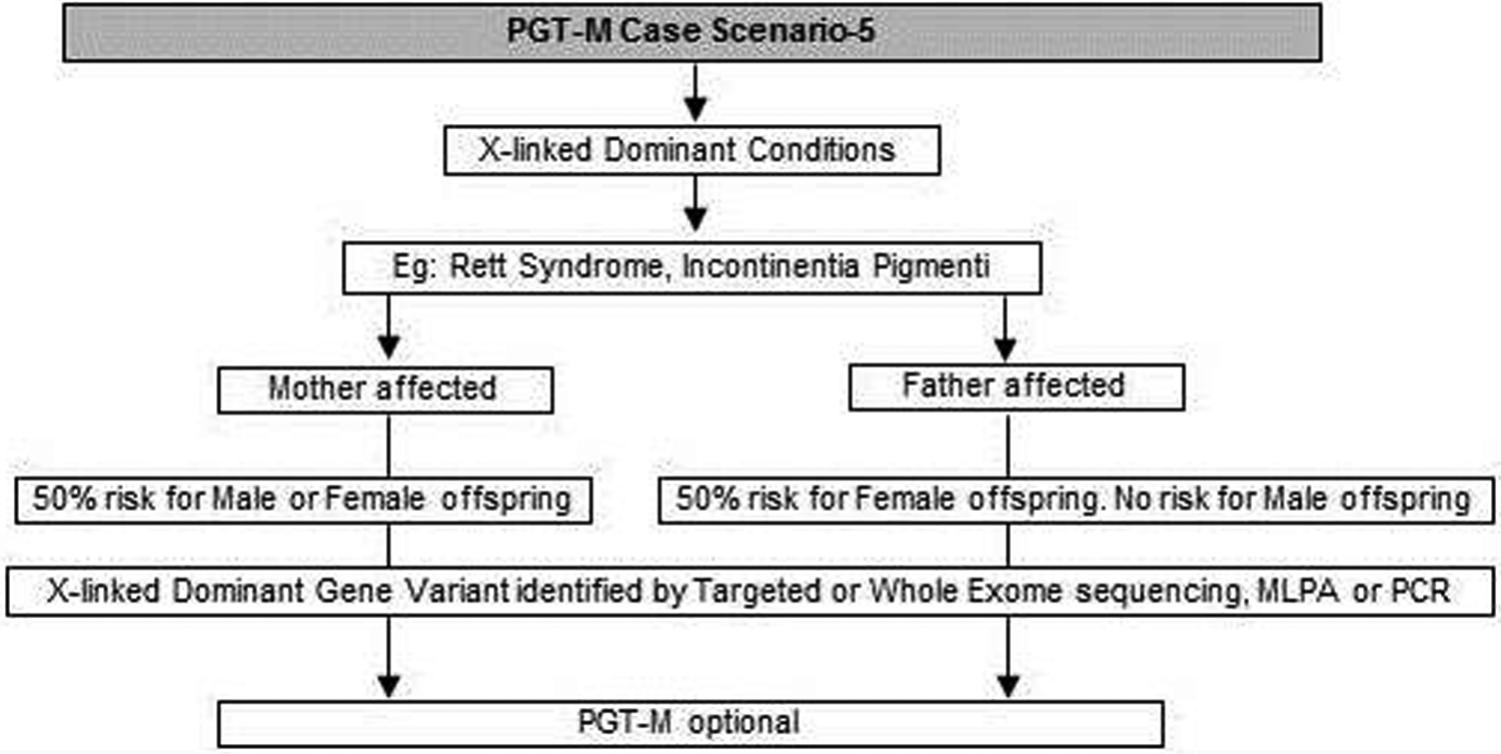
Genetic diagnostic workflow for PGT-M of X-linked dominant conditions.

Most of the Mendelian disorders are caused by disease causing variations in a single gene (monogenic). The inheritance of these conditions can be autosomal dominant, autosomal recessive, X-linked dominant or X-linked recessive. The involvement of the genetic counselor begins by looking at the phenotype of the affected individual in the family, doing a dysmorphology assessment, reviewing the medical records, determining the inheritance pattern and ordering tests to identify the disease-causing variation along with its inheritance. Based on the phenotype analysis, the counselor follows a genetic diagnostic workflow which is economical and best suited for the case (Figures 1–5). The following case examples demonstrate the adopted workflow in frequent counseling scenarios:

(a) Common genetic variations such as ΔF508 in cystic fibrosis [c.1521_1523del (p.Phe508del)] and c.20A>T (p.Glu6Val) in sickle cell anemia can be checked by simple molecular techniques such as polymerase chain reaction (PCR) (24–27). If the variation is confirmed either in the index case or the carrier parents, PGT-M can be offered for the condition (Figure 1).
(b) If common genetic variations are absent in a case with clear cystic fibrosis or sickle cell anemia phenotypes, the genetic counselor will order tests with wider scope such as full gene sequencing of *CFTR* or beta globin gene to identify rare pathogenic variations (28, 29). Many times it is seen that the individual with sickle cell anemia may actually have sickle cell thalassemia with a sickle cell heterozygous variation and a thalassemia heterozygous variation (30). Full gene sequencing will help to identify such cases. Based on these results, the option of PGT-M can be discussed with the family (Figure 1).
(c) In autosomal dominant conditions such as history of a child with achondroplasia, confirmed by a genetic test, the recurrence risk is usually low as parents are unaffected. The sporadic occurrence of achondroplasia and low recurrence risk is explained to the couple and PGT-M may not be offered. The extreme rare condition of gonadal mosaicism, where parents may be unaffected carriers but produce gametes with a variation due to the presence of a variation only in gonadal tissue should be discussed. In such condition PGT-M can be offered after counseling. However, in a homozygous state, achondroplasia is usually embryonically lethal (31) (Figure 2).
(d) If a parent has an early onset autosomal dominant Alzheimer, Huntington disease or Marfan syndrome, the risk of recurrence for offspring is 50% hence the PGT-M option is explained to the couple (32–34) (Figure 2).
(e) For autosomal recessive disorders, the workflow will include identification of a recessive variation in the index child or identification of the carrier status in both partners (35, 36). Examples of recessive disorders include thalassemia, cystic fibrosis and albinism (37). A recurrence risk of 25% for every pregnancy is discussed with the family during the counseling session based on the confirmation of a recessive variation in the index case or carrier status of both partners by genetic tests. In some instances where the proband or affected family member is unavailable, carrier screening is recommended for parents to identify the general risk of inheriting recessive disorders that are not indicated by family history. Carrier screening can be offered for selective targeted disorders by targeted NGS panels or for all recessive disorders using whole exome sequencing (WES) methodologies. This testing is particularly helpful in consanguineous marriages. Genetic counseling in the presence of consanguinity is vital in order to explain the possibility of more than one recessive disorder risk in the offspring because of the higher proportion of shared genetic material between the partners (38) (Figure 3).
(f) In cases such as Duchene muscular dystrophy (DMD) or spinal muscular atrophy (SMA), the multiplex ligation dependent probe amplification (MLPA) technology is utilized to identify exon level deletions or duplications. Confirmation of pathogenic homozygous deletion/duplication in affected individuals or hemizygous/heterozygous deletion/duplication in carrier parents will open up the discussion on the option of PGT-M (Figure 4) (39, 40).
(g) For X-linked recessive conditions like hemophilia, where males are mainly affected, the counselor will recommend identification of the gene variant in the affected male child or in the female partner, who is usually an unaffected carrier with a variation on one of her X chromosomes, the other X chromosome being normal. A 50% risk to the male offspring is explained as the risk that she passes on when she transmits an X chromosome with the variant. Female offspring are usually unaffected like their mother (41) (Figure 4).
(h) In certain scenarios the counseling session will clarify that there is no risk to the offspring and PGT-M may not be required. For instance, if the male partner's brother has DMD which is an X-linked recessive condition, there is no risk for the couple as the male partner has a normal phenotype being devoid of the variation on his X chromosome (Figure 4).
(i) Although rare, X-linked dominant conditions such as, Incontinentia pigmenti and Rett syndrome equally affect males and females. Males with X-linked dominant condition are affected more severely with low survivability than females. In many cases *in-utero* demise is a common phenomenon observed in affected male fetuses. An affected mother will have a 50% chance of passing on the dominant gene variant to either male or female offsping. In case of an affected father there is 50% risk for female offspring and no risk for male offspring as the father does not contribute the X-chromosome to a male child (Figure 5).
(j) There are many other complex disorders with multi-gene etiology and for their diagnosis including differential diagnosis, NGS based WES is used. The test relies on sequencing of genomic exons or the coding regions of the genome and is usually recommended for the affected family member (Index or proband) (42, 43). Couple carrier screening can also be offered. Based on the test results, the genetic counselor would guide the family and patients for follow-up tests which could be either in the prenatal or pre-implantation period.
(k) There are many disorders for which PGT-M was successfully offered (44–46).

## The dilemma of variants of uncertain significance (VUS)—pathogenic or benign?

With the advancement of NGS technologies allowing rapid and inexpensive sequencing of the human genome, we are witnessing its rapid adoption in clinics for couples as a preliminary genetic work up by choice, before starting any fertility treatment. A change in the genetic code is usually called a variant and thousands of variants are identified in the process of WES or whole genome sequencing. These variants are classified into five tiers: pathogenic (P), likely pathogenic (LP), variant of uncertain significance (VUS), likely benign (LB), and benign (B) as per ACMG guidelines. Pathogenic and likely pathogenic variants are those that are strongly associated with disease etiology hence offering PGT-M in such situations is justified. Benign and likely benign are those variants that may not be disease causing. Therefore, PGT-M is not offered. Variants that are not classified either as pathogenic or benign are reported as VUS (21). A VUS is a genetic change whose clinical impact is not yet well understood, either because its effect on the gene's function is not fully known or because there is lack of sufficient data for its definitive classification as benign or pathogenic due to a low number of cases reported or due to conflicting results in literature. While many laboratory policies reject PGT-M requests for VUS, some labs accept them, provided the patients clearly understand the implications of testing the VUS (47). An in-depth counseling session will help couples understand these details and a clear documentation of the same communication between the genetic counselor and patients should be made available to the lab along with the signed consent form. It should be noted that the classification of a variant may change over time to likely pathogenic or likely benign with increasing availability of data on the gene variant. Laboratories providing sequencing and reporting services may contact families if there is a change in classification of the variant and issue a revised report. Patients should be encouraged to attend a genetic counseling session with a revised report. Sometimes testing extended family members can help in resolving the issue of pathogenicity of the VUS. This is done by determining whether variants are shared by other affected or unaffected individuals. However, this requires a prolonged work-up of multiple family members and may not be practical in all cases. Considering the above issues with VUS, the focus of the genetic counselor should be to address the uncertainty in such a way that the couple understands the implications in order to make an informed decision.

## The process of pre-PGT-M workup

PGT-M optimization includes designing laboratory primers and pre-work-up using DNA of extended family members. The laboratory usually carries out a linkage-based analysis using multiple genetic markers on parents and available family members before testing the embryos. In some cases, the genetic counselor may involve more team members including laboratory personnel and clinical experts in the discussion in order to determine the feasibility of testing the embryos (48). PGT-M may sometimes be complex and difficult in cases of *de novo* variants, unconfirmed VUS in extended family members, unavailability of relatives, refusal for testing by relatives, in the presence of complex gene loci and other technical difficulties.

## Genetic counseling for mitochondrial disorders

Mitochondrial disorders mostly result in metabolic problems. They involve multiple organs with variable disease onset and severity. Variations in both nuclear DNA and mitochondrial DNA (mtDNA) are responsible for mitochondrial disorders.

Disorders caused by variations in nuclear DNA usually follow an autosomal recessive pattern of inheritance, although rarely an autosomal dominant pattern is seen. Prenatal diagnosis and PGT are available to prevent the birth of another severely affected child in such cases of nuclear gene associated mitochondrial disorders (Variation in *SURF1* gene for Leigh Syndrome) (49). For *de novo* variations, the recurrence risk is low and couples can be counseled accordingly.

However in 15%–25% of cases, mitochondrial diseases are caused by mtDNA variations, which can be either *de novo* or maternally inherited. Mitochondrial DNA sequencing is used to confirm the variations. But for maternally inherited mtDNA variations, the recurrence risk is often unpredictable because of random presence of heteroplasmy (a mixture of normal and variation copies in mtDNA) or homoplasmy (all copies of the mitochondrial genome are identical with or without the variation) in cells. In addition, mtDNA variations are associated with a variable phenotype which leads to difficulty in predicting the phenotypic outcome. The option of oocyte donation may be discussed to prevent transmission of the disorder to the offspring. Discussion of complex mitochondrial inheritance needs extended sessions during genetic counseling (50). Recently, mitochondrial replacement therapy (MRT) has become available for clinical application as an alternative to prevent the transmission of heteroplasmic and homoplasmic mtDNA variations (51).

## Genetic counseling for inherited cancer predisposition syndromes

Individuals are considered to be candidates for inherited cancer predisposition syndrome risk assessment if they have a personal and/or a family history on the maternal or paternal side or if they have clinical characteristics with features suggestive of inherited cancer predisposition syndrome. Some examples are familial breast and ovarian cancer, Lynch syndrome, Peutz–Jeghers syndrome and inherited retinoblastoma. Testing the affected individual or an individual at risk is essential to determine the inheritance pattern and identify the risk for future generations. Although prenatal and pre implantation testing can be offered to families at a high risk, it must be kept in mind that testing is done for germline cancer susceptible variants that increase the risk for malignancy and not for somatic variations. PGT can limit the transmission of the inherited cancer gene variation to future generations (52–55). It is essential to discuss the challenges that may be encountered including the age of onset, variable penetrance and phenotype variability. Both pre-test and post-test counseling sessions are recommended, so that the individuals are made aware of the details of the genetic report's cancer predisposition for appropriate planning of PGT-M.

## Genetic counseling for adult-onset genetic disease

PGT for late adult-onset diseases is complex with a wide range of ethical, legal, social, and policy issues. PGT-M for late adult-onset conditions is a controversial subject with debatable arguments on both sides. The Ethics Committee of the American Society for Reproductive Medicine (ASRM) states that PGT-M for adult-onset conditions is ethically justified when the condition is serious, and no safe, effective interventions are available. The committee however cautions about technological issues, complexity of the scientific, psychological and social aspects and emphasizes on the strong role of an experienced genetic counselor in the process (56). Examples of adult-onset genetic conditions are Huntington disease, myotonic dystrophy, spinocerebellar ataxia, Charcot-Marie-Tooth disease, adult-onset metachromatic leukodystrophy, neurofibromatosis and hereditary cancer predisposition syndromes. While patients with a positive family history seek to understand their carrier status for late onset diseases, sometimes the associated variants are identified in WES tests as secondary or incidental findings. Labs disclose such information only to individuals who consent to know their risk. Once the risk is identified, the adult may choose not to pass on the variant to the offspring and might seek PGT-M for the condition. Many labs accept such a request with the individual's consent. However, one important aspect to consider when discussing PGT-M for late adult-onset genetic diseases is the issue of penetrance. If some individuals with the variant do not develop features of the disorder while others do, the condition is said to have reduced or incomplete penetrance. In such cases, ethical and policy considerations including reproductive liberties take a central place in the discussion. If the variation is identified in an affected grandparent, the children in the reproductive age group could get pre-symptomatic testing done with a view to avoid passing on the deleterious gene to the next generation. Occasionally, these couples may not want to know the result of the pre-symptomatic test, but may want to do PGT anyway, in order to avoid transmission of the pathogenic variant to their children. This is feasible and can be done after discussing non-disclosure protocols and taking appropriate informed consent. The genetic counselors must consider all such situations and provide information to patients in order to facilitate non-directive decision making (57, 58).

## Genetic counseling for polygenic disorders (PGT-P)

Polygenic diseases are complex diseases that are influenced by the combined effects of many genes along with an environmental contribution. Examples include commonly occurring diseases, such as coronary heart disease and type 2 diabetes, inherited cancer predisposition syndrome and schizophrenia. Unlike PGT-M, PGT for polygenic disease (PGT-P) represents a further level of complexity in which multiple genes are tested and an associated polygenic risk score (PRS) is established using genome-wide association studies (GWAS). PRS estimates the genetic risk of an individual for a disease or trait, calculated by aggregating the effect of many common variants associated with the condition. Assigning a PRS to an embryo is possible because of available large datasets of people with each disease. Clinical implementation of PRS may be useful in cohorts where there is a higher prior probability of disease, for example, in early stages of diseases to assist in the diagnosis or to inform individuals regarding treatment choices (56, 59–62). The pros and cons of PGT-P are highly debated among the scientific circles and there are vocal proponents and opponents on both sides arguing cost to benefit justification (63). Although a few companies are already offering such services, genetic counselors should caution families of the limitations such as uncertainty of the risks, non-availability of data for many population groups, absence of long term studies and the fact that it is only predictive with a risk score and not diagnostic. Currently these limitations lead to attenuated feasibility of prenatal or PGT-P (64).

## Genetic counseling for epigenetic disorders

The epigenome surrounds the genome or the genetic component of the cell and influences the mechanism of turning on and off the genes by condensation, folding and unfolding of DNA. Histones, methyl groups and microRNAs constitute the epigenome. Many human diseases including neurodevelopment conditions, cancer and life style disorders are influenced by epigenetic changes which are dependent on the environment. Although the search for new fetal epigenetic markers and the clinical implementation of epigenetic approaches for noninvasive prenatal diagnosis are underway (65), prenatal and PGT options are not currently feasible. Pre-pregnancy parental lifestyle, post pregnancy maternal care and early childhood nurturing, make for an optimal epigenome that can protect individuals throughout their life span (66).

## Post PGT-M genetic counseling

**Figure 6:**
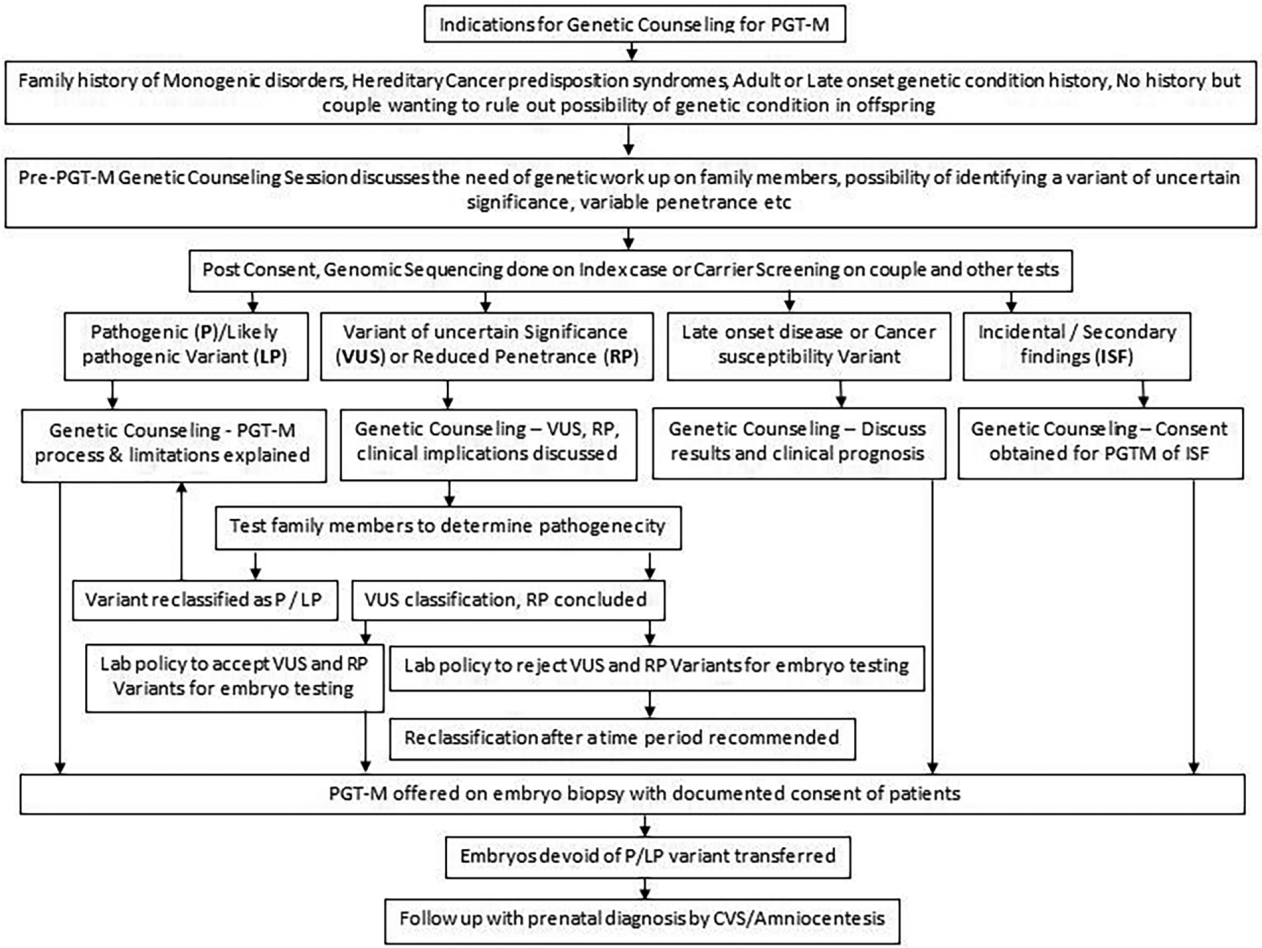
Summary of end to end genetic counseling work flow for PGT-M.

A post-test genetic counseling session explaining the PGT-M results and their interpretation should be carried out. Usually, along with testing for the monogenic disorders, PGT-A is also offered on the embryos so that the risk of aneuploidy is also ascertained (48). Aneuploid embryo with a normal or unaffected carrier status for the tested genetic condition is chosen for transfer to the uterus at a time when the endometrium is most receptive. Other normal embryos are cryopreserved for future use. It is important to counsel individuals about the rare false negative results, however rare, caused by both technical limitations and complex biological processes. For example, a disintegrated DNA sample might fail to amplify both the alleles of the variant simultaneously, resulting in a false homozygous report. This phenomenon of amplification of only one allele, instead of both, is called as allele drop-out. *In vivo*, the early embryonic divisions are prone to errors and may give rise to sporadic genetic variants that may be pathogenic in the fetus. Hence diagnostic prenatal tests by chorionic villus sampling (CVS) or amniocentesis processes should be communicated during counseling in a simple language with emphasis on the need of post-conception testing. The entire process of genetic counseling in PGT-M is summarized in a flow chart in Figure 6. It gives a broad outline of steps usually followed by the genetic counselor before initiating the tests and after evaluating the type of result (pathogenic, likely pathogenic, uncertain significance variants), late onset disease diagnosis and secondary findings.

## Genetic counseling for PGT-M for a savior sibling

A savior sibling is usually considered by parents when a child needs a bone marrow transplant and finding a compatible donor is a challenge. The savior baby is generally created through IVF by testing the embryos by human leukocyte antigen (*HLA*) typing and using the embryos that are 100% *HLA* matched with the older affected sibling. A wide range of genetic disorders can be treated by savior siblings including blood disorders like thalassemia, leukemia, bone marrow disorders, immune deficiencies and certain cancers. It is important to select an embryo which is not only 100% *HLA* match but is also devoid of the pathogenic disease variation present in the older sibling. Haematopoietic stem cells collected from the umbilical cord blood or the bone marrow of the *HLA*-matched donor sibling born are used for transplantation to cure the affected sibling (67). *HLA* typing of ART-created embryos was first reported in 2001 (68) and with several encouraging results reported worldwide, savior sibling creation is now offered in several IVF clinics (69). The genetic counselor will have to discuss ethical, technical and financial implications of a savior sibling during the genetic counseling session (70).

## Conclusion

The role of genetic counseling for couples undergoing PGT-M is crucial and must be offered by trained, qualified genetic counselors. Usually the information gathered during a genetic counseling session helps the counselor to not only offer PGT-M option based on the case details, but also to select appropriate technology or workflow for pre-PGT-M work up. Genetic counselors will have to consider the inheritance pattern, availability, or non-availability of index case, family history and clinical parameters before giving out the recommendations. Complex genetic disorders including mitochondrial diseases and polygenic conditions must be addressed with care. Communicating genetic test results to the family and helping them understand the implications is an important part of the sessions. The genetic counselor plays a pivotal role in establishing a close collaboration between genetic labs and ART centers which is paramount for successful implementation of PGT-M. As couples would be going through complex IVF treatment together with genetic testing, it is expected that they may be sensitive and emotionally charged and may ask abundant questions. A genetic counselor should be proficient with the language of the patient/family, have empathy, should be willing to listen and explain, be supportive, non-directive, and use effective communication through charts and videos. Genetic counselors should align their discussion with ethical, religious issues and the rules and regulations of the governing body of the residing country.
